# The impact of a virtual wound on pain sensitivity: insights into the affective dimension of pain

**DOI:** 10.3389/fpain.2025.1502616

**Published:** 2025-02-26

**Authors:** Ingrid Koopmans, Robert-Jan Doll, Maurice Hagemeijer, Robert van Barneveld, Marieke de Kam, Geert Jan Groeneveld

**Affiliations:** ^1^ Centre for Human Drug Research, Leiden, Netherlands; ^2^ Clinical Pharmacology, Leiden University Medical Center, Leiden, Netherlands; ^3^ Righteous Games, Eindhoven, Netherlands

**Keywords:** virtual reality, pain, affective component, drug development, biomarker

## Abstract

**Background:**

The perception of pain is difficult to assess due to the complex combination of various components related to nociception, experience, and cognition. There are currently no biomarkers to assess the affective component of pain in healthy volunteers. Using Virtual Reality (VR), it may be possible to assess changes in pain perception when adding an affective component to painful stimulation.

**Methods:**

In this two-visit feasibility study, we assess the effect of a simulated wound in VR on the electrical pain detection (PDT) and tolerance (PTT) threshold in 24 healthy male study participants. The VR simulation presented a copy of the research room from first person view. Prior to each VR assessment, study participants were primed by interacting with the VR environment. Two conditions were assessed: (1) VR-Wound: a burn-wound, smoke, and electrical sparks become visible and audible with increasing stimulus intensity, and (2) VR-neutral: no additional aspects. The PDT and PTT to electrical stimuli were recorded during both VR conditions and outside of VR. VAS-Questionnaires were used to assess unpleasantness and fear.

**Results:**

The PDT decreased when a virtual wound is presented compared to a neutral condition. Study participants experienced the electrical stimulation as more painful and more intense during the wound simulation than during the neutral condition. The effect was more pronounced during the second visit.

**Conclusion:**

VR enhanced the perception of pain, thereby providing new insights into the affective component of pain. Further testing of this methodology is warranted by performing a clinical study that evaluates drug effects on the affective component of pain.

## Introduction

The affective component of pain plays an important role in pain and is linked as an important factor to cases of chronic pain (1, 2). Emotions can modulate the experience of pain. However, singling out the affective component of pain in a clinical research setting remains difficult (3, 4). As a result, demonstrating that a (drug) treatment is effective at alleviating pain by addressing the affective component is challenging (1, 2).

The analgesic effects of new drugs are commonly assessed in early phase clinical drug studies using various well-established tests, and are ideally conducted in healthy study participants. These tests are particularly valuable if they provide early indications of a drug's efficacy, which is strongly dependent on the availability of pharmacodynamic biomarkers to be used for proof of pharmacology, proof-of- mechanism, or proof-of-concept. Analgesic effects in healthy study participants can be assessed by changes in pain detection thresholds (PDT) and pain tolerance threshold (PTT) to stimuli (e.g., electrical, heat, or pressure) (5). To attribute the effect of the change in threshold to the studied intervention, these tests are performed in a controlled environment, minimizing external interferences and distraction. However, healthy study participants in this setting will unlikely show sufficient variation in the affective component of pain—without being challenged-, making it difficult to study the effects of analgesic compounds that target the affective component in an isolated fashion. By adding a challenge that adds an affective component to a pure nociceptive task, the task becomes more susceptible to the effects of new analgesic compounds that target pain syndromes in an affective pain component plays an important role. Such a task may produce a suitable pharmacodynamic biomarker, which can be used in early phase clinical drug studies of analgesics influencing the affective component of pain.

It is well known that the perception of pain can be altered due to distraction or anxiety. When distracted, both children and adults report less pain (6, 7). In contrast, inducing anxiety can increase pain intensity and unpleasantness (8). Interpreting a (painful) stimulus as potentially harmful influences the reported levels of pain (9). Additionally, creating an illusion for the study participants within reasonable limits, such as the rubber arm paradigm is found effective suggesting threat without a nociceptive stimulus (10). It therefore seems clear that it is possible to modulate pain experience in a controlled pain experiment. A promising possibility to modulate a person's pain experience by a combination of focus and anxiety might be by using Virtual Reality (VR).

Current research on VR in relation to pain is primarily focussed on alleviating the perception of pain by deep immersion in a distracting setting (11, 12). Others have studied the fundamental aspects of the effect of VR on pain. For example, it was demonstrated that the level of virtual ownership of an avatar (simulated person) affects the pain experience (13–15). The simulated size of affected body parts and transparency of these body parts also influence pain experience (16, 17). Using VR to introduce a coloured area on the location of a painful stimulus was demonstrated to be effective to modulate the pain experience (17). Due to the used heat paradigm, PTT recordings were not feasible due to the risk of skin damage. Another study including a burning hand simulation in augmented reality also showed a reduction in PDT (18). Pain experience questionnaires, which is the current standard for emotional responses on pain, are not yet included in a study with VR.

A VR simulation with a realistic visual enhancement of consequences of the stimuli combined with audio related to the pain experiment has never been performed. In this study, we combine an electrical pain test with VR. In VR, the electrical stimulation is accompanied with sounds and visuals of electrical sparks, and an increasingly damaging skin underneath the stimulating electrodes. With this, we aim to add an affective component to a nociceptive stimulus with the purpose to try to exacerbate the pain in a setting closer to real life. In addition to capturing the pain detection and pain tolerance thresholds, qualitative aspects (e.g., subjective scales for anxiety and fear, and personality questionnaires) were also recorded. This setup could potentially provide biomarkers (pain thresholds) to study effects of analgesic drugs that target the affective component of pain.

## Methods

This was an exploratory single-centre two-visit cross-over study. The study was conducted between March and July 2021 at the clinical research unit of the Centre for Human Drug Research (CHDR) in Leiden, The Netherlands. The study was approved by the Medical Ethics Committee Stichting Beoordeling Ethiek Biomedisch Onderzoek (Assen, the Netherlands). Study conductance was according to the Dutch Act on Medical Research Involving Human Subjects (WMO) and in compliance with all International Conference on Harmonisation Good Clinical Practice (ICH-GCP) guidelines and the Declaration of Helsinki. The study was prospectively registered in the International Clinical Trials Registry Platform as NL-OMON28178.

### Study design

**Figure 1:**
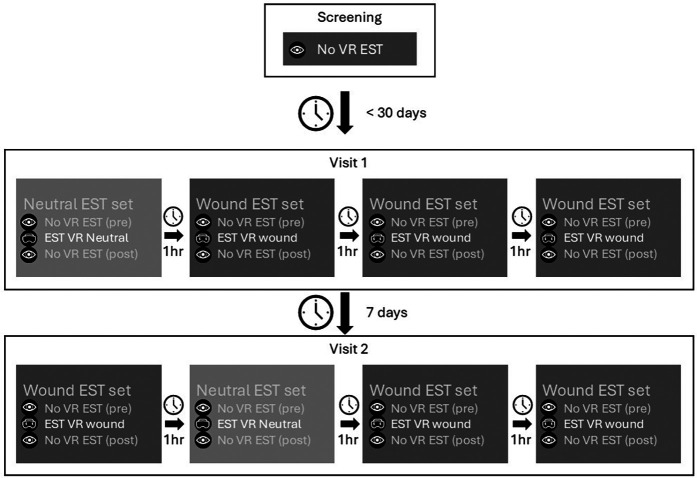
Schematic overview of study design and the assessments. The questionnaires are left out of the figure for clarity. Each set contained the following assessments: pain task without VR—VAS pain—STAI-6—VAS fear—pain task with VR (either neutral or wound)—VAS pain—STAI-6—VAS fear—embodiment—pain task without VR—VAS pain.

Potential study participants underwent medical screening and training on the pain task without VR. In this same visit, study participants filled in the personality questionnaires as described below. After inclusion, the study consisted for each study participant of two visits, each one day starting at around 9:00 and finalized around 16:00. Study participants were admitted to the clinical research unit for the duration of the visit and discharged after completion of all study assessments. A rest period of 7 days was included in between both visits. All study participants underwent the same procedures during each visit. Each visit started with a urine drug test and alcohol breath analysis after which the anxiety inventory was performed. During the study visit, four sets of pain tasks were completed. One set of pain tasks included one VR assessment (either with wound or neutral) and a before and after assessment without VR. This to function as a baseline and to potentially capture the long-term effect of the VR simulation. A total of 12 pain tasks were completed for each study visit. The first visit started with the neutral VR simulation followed by three sets of assessments with the wound simulation. The second visit also contained three sets of pain tasks with the wound but had the neutral simulation included in as second set. The assessments are repeated during each visit to increase the power of the study. After each assessment, study participants reported the pain experience using VAS questionnaires. A rest period of 1 h separated each set of assessments. See Figure 1 for a schematic overview of the study activities.

### Study participants

All study participants provided written informed consent prior to undertaking any study-related activities. To match the avatar in VR, only healthy male study participants between 18 and 40 years of age were invited to participate. Only light to medium skin tones (Fitzpatrick ≤IV) were allowed and no deformations or (dis)colouring of the skin was allowed in upper and lower limbs. Study participants with a pain tolerance threshold >80% of the maximum stimulation of the test (without VR) were excluded in the study. No history of psychiatric illness or visionary disorders were allowed. Study participants who smoked more than 5 cigarettes per day on average or consumed more than 8 units of (methyl)-xanthine a day were excluded because of possible withdrawal symptoms during study participation. Study participants who had previously experienced Simulator Sickness Syndrome with either VR or another simulator were not eligible to participate. During screening, study participants were neither trained on the VR simulation nor given information about the content of the VR simulation. The sample size of this study was not based on a formal sample size calculation due to the exploratory nature. As it is our aim to use this method in early phase clinical drug studies we chose a sample size that is typically used in in early phase clinical drug studies of analgesics.

### Assessments

All measurements were performed in a quiet room. During all assessments only the study participant and a research assistant was present in the room. To prevent infection with Covid-19, all study participants wore face masks throughout the study and the equipment was cleaned with disinfectant in between study participants.

#### Participant personality characteristics

##### Temperament and character inventory

The Temperament and character inventory (TCI) was developed by Cloninger et al. and widely accepted for personality assessments (19, 20). The TCI contains 240 items which needs to be answered with “correct” or “incorrect”. The Dutch translation was provided in digital form by Datec and used during the screening visit. Endpoints include seven dimensions of temperament and character: Novelty seeking (NS), harm avoidance (HA), reward dependence (RD), persistence (PS), self-directedness (SD), cooperativeness (CO) and self-transcendence (ST). Each of these dimensions are divided in multiple sub-factors resulting in a total of 24 subscales.

##### Pain catastrophizing scale

Dutch language version of the Pain Catastrophizing scale (PCS) evaluates the pain-related thoughts and emotional distress related to pain (21, 22). The questionnaire consists of 12 self-report questions with a 4-point scale measuring three components of catastrophic thinking: rumination, magnification, and helplessness. The PCS is only performed at screening.

##### Pain-anxiety symptoms scale

The Pain-Anxiety Symptoms scale short form (PASS-20) was presented to the study participant during the screening visit (23). The questionnaire consists of 20 self-report questions with a 5 Likert-scale measuring four dimensions of pain-related fear and anxiety: Cognitive anxiety responses, escape and avoidance, fearful thinking, and physiological anxiety responses. The PASS-20 is only assessed during the screening visit.

##### Spielberger state-trait anxiety inventory

State and trait Anxiety was measured with the Spielberger State-Trait Anxiety Inventory, trait scale (STAI-DY) (24). The STAI-DY consists of 40 questions with a 1–4 scale: 20 items are related to the State-Anxiety (STAI-DY-1) and 20 items are related to the Trait-Anxiety (STAI-DY-2) subscales. The STAI-DY-1 is assessed both during the screening visit and at the start of each study visit. The STAI-DY-2 is assessed only during the screening visit.

#### Electrical stair test

The electrical stair test uses two electrodes (Ag-AgCl) on the tibial bone to assess cutaneous electrical pain. Single electrical stimuli are provided with a duration of 0.2 ms, increasing from 0 mA to a maximum of 50 mA in steps of 0.5 mA. Study participants were provided with an electronic version of the Visual Analogue Scale (eVAS) and instructed to start moving the slider when the stimulus became painful. The intensity of this pain detection threshold (PDT) is the first endpoint of this pain task. The second endpoint recorded is the pain tolerance threshold (PTT), the intensity at which the study participant indicates the maximum value on the eVAS which corresponds to the maximum pain tolerated. If the study participant does not indicate the PTT before 50 mA, the maximum duration of the test is 120 s after which the stimulation is stopped automatically.

After each electrical stair test, four electronic VAS assessments were used to evaluate the level of pain, unpleasantness and intensity of pain, and fear.

#### Virtual reality

##### Equipment

Study participants wore a VR headset with headphones (Vive Pro, HTC) during the VR-Pain measurement. The VR environment emulates the room in which study participants performed all assessments. The VR includes an avatar of the study participant from a first-person view. The chair and equipment of the electrical stair pain test (including the electrodes on the leg and a VAS slider) were included as well. To ensure embodiment, the position of the legs, hands, and VAS slider were tracked using HTC Vive trackers and a leap motion sensor. Additionally, the skin colour of the avatar matched the most frequently occurring skin colour in the Netherlands (i.e., Fitzpatrick II–III).

##### Priming and perception of embodiment

Prior to each VR assessment, study participants were primed by performing a set of instructions encouraging interaction with the VR environment. The instructions included asking the study participant to grab the VAS slider from the sky (handed by the assistant) and describe objects located in the room. The study participant controlled both the start and stop of the test, including the simulation, using the VAS slider.

After each VR assessment, the study participants' perception of embodiment was evaluated. Six statements related to embodiment were presented to which could be answered using a 7-point Likert scale (1: completely disagree, 7: completely agree). The statements were: (1) the virtual body parts felt like my own body parts, (2) it felt like the virtual body was my own, (3) when I saw the wound appearing on my leg it felt like the wound was a part of me, (4) the movements of the virtual body appeared like my own movements, (5) I felt I had control over the movements of the virtual body, and (6) I had the illusion owning a different body than my own.

##### VR conditions

**Figure 2:**
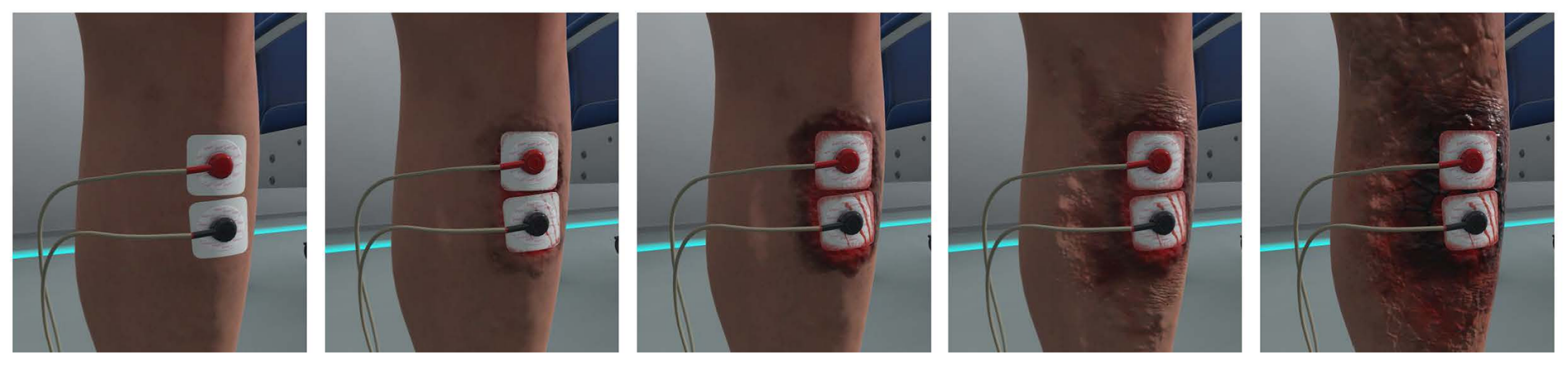
Virtual reality simulation of the burn wound on the leg increasing in severity from left to right.

There were two different VR conditions: (1) VR-Wound and (2) VR-Neutral (see Figure 2). The VR-Wound condition shows a burn wound around the electrodes on the leg. The intensity of the wound increases simultaneously with the intensity of the pain test. This simulation is accompanied by sounds of electrical sparks through the VR headset. The simulation started directly at the beginning of the test and reached maximum intensity at 40 s. After 40 s, the intensity of the audio-visual simulation no longer increases but continues until the test is stopped. This to make sure most of the study participants experience the full simulation. During the VR-Neutral condition, no additional visual or auditory stimulations were applied.

Subjects were instructed to look at the electrodes which was monitored by the research assistant via a mirror image of the VR view on the computer. It was not possible to confirm if the subjects had their eyes open during the assessment. After each assessment including the VR-wound simulation, study participants scored the simulation on realism, unpleasantness, and their focus on the wound during the pain task.

#### Statistical analysis

The statistical analysis was preceded by a data review which consisted of visual inspection of individual graphs per visit of all efficacy measurements by time. To establish whether significant effects can be detected on the repeatedly measured pain parameters, the change from baseline of each parameter was analysed with a mixed effects model with condition (pre VR, VR neutral, VR wound or Post VR), visit (day 1 or day 2), session (1, 2, 3, or 4 within day), condition by visit, condition by session, visit by session and condition by visit by session as fixed factors and study participant, study participant by visit and study participant by session as random factor. The Kenward-Roger approximation was used to estimate denominator degrees of freedom and model parameters were estimated using the restricted maximum likelihood method.

The TCI was compared with external data using a two-sided *t*-test, as was the difference between baseline assessments with the STAI on each visit.

## Results

### Study participants

A total of 25 healthy male study participants were enrolled in the study. One study participant stopped participation after the first visit due to Covid-19 quarantine requirements in the Netherlands. He was replaced and excluded for statistical analysis. The other 24 study participants were included for statistical analysis [age mean (SD) is 23.3 (5.0), range 18–34]. No relevant datapoints were excluded based on the blinded data review. Data could not be collected for two VR measurements of separate study participants due to technical difficulties and two VR measurements of two other study participants were lost because of an emergency evacuation practice drill of the clinical research unit. Additionally, answers to related questionnaires for these measurements were not collected.

### Effects of virtual reality

#### Pain thresholds electrical stair task

**Figure 3:**
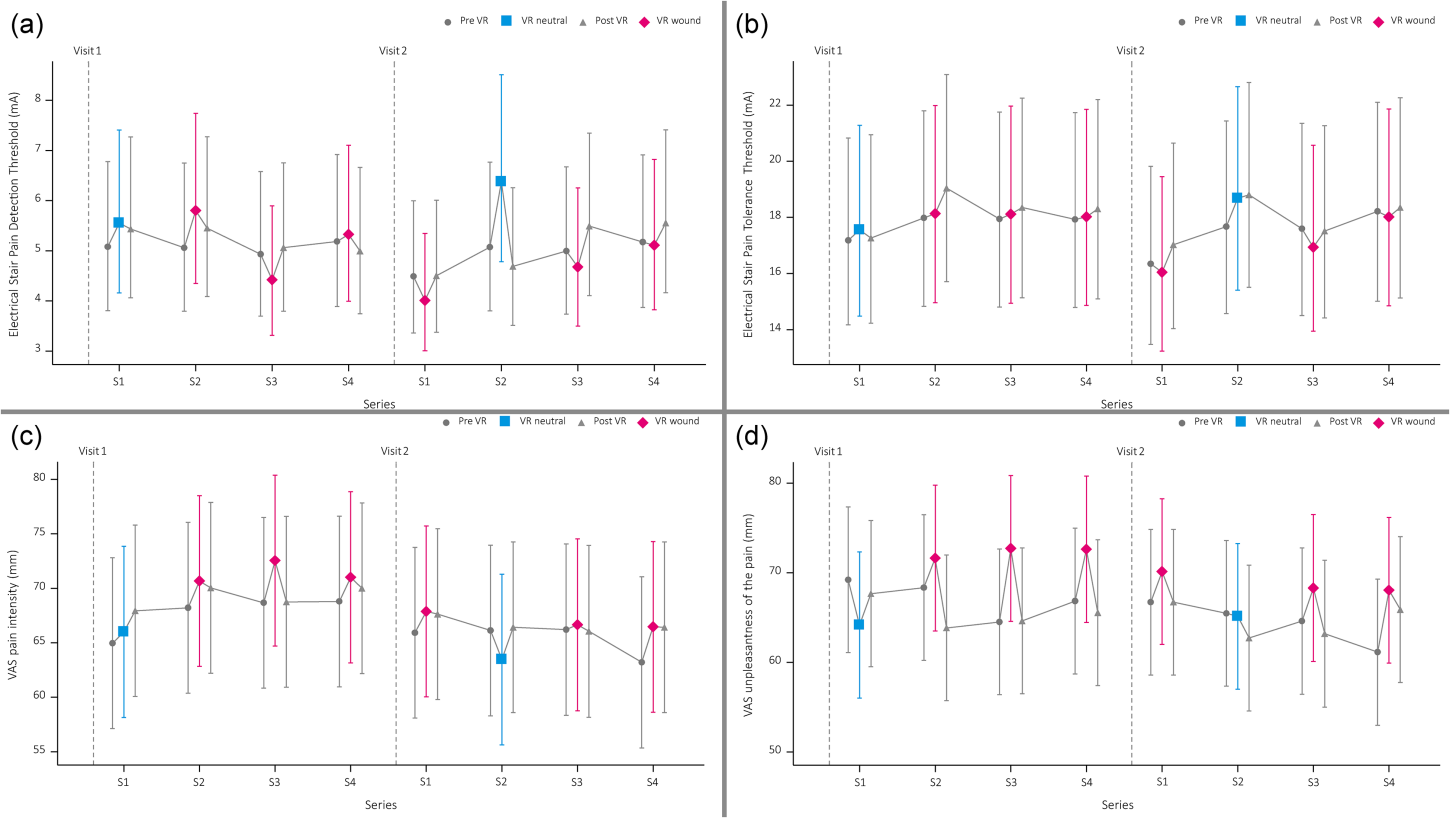
Graphical overview of pain detection threshold **(a)**, pain tolerance threshold **(b)**, visual analogue scale (VAS) score of the pain intensity **(c)** and experienced unpleasantness of the painful stimuli **(d)**.

The least square means of all PDT and PTT values are presented in Figure 3. The mean PDT during the VR Wound condition (4.85 mA) was significantly lower [−18.4%, 95%CI: (−26.9%, −9.0%) *p* < .001] than the PDT during the VR neutral simulation (5.95 mA). This was more pronounced during the second visit, see Figure 3. The pre-VR neutral measurement was significantly lower [17.2%, 95%CI: (3.0%, 33.4%) *p* < .016] compared to the VR neutral simulation (5.08 mA and 5.95 mA, respectively).

For the PTT, no statistical difference was found between the VR neutral and VR wound simulation (18.11 mA vs. 17.52 mA, *p* = .21). Additionally, no significant difference was observed for the PTT between de pre-VR neutral measurement and the VR neutral test (17.42 mA vs. 18.11 mA, *p* = .21). However, there was a significant difference for the PTT between the VR neutral (18.68 mA) and VR wound (16.04 mA) simulation for the second visit [−14.1%, 95%CI: (−21.5%, −6.1%) *p* < .001].

#### Pain perception during electrical stair task

Study participants rated the pain during the VR wound simulation (70 mm) significantly more intense [4.5, 95%CI: (1.8, 7.2) *p* = .0013] compared to the VR neutral simulation (65 mm). Additionally, the pain was rated significantly more unpleasant [5.9, 95%CI: (2.1, 9.8) *p* = .0028] for the VR wound simulation (71 mm) compared to the VR neutral simulation (65 mm).

The pain intensity and unpleasantness were not scored significantly different between the pre-VR pain test and the VR neutral pain test [intensity: 95%CI: −0.8 (−4.0; 2.4) *p* = 0.6, unpleasantness: 95%CI: −2.7 (−6.8; 1.4) *p* = 0.2]. See Figure 2 for an overview.

#### Embodiment and subjective experience of the wound simulation

The level of embodiment during the VR simulations was for both the neutral and the wound simulation on average 21.73 points with a standard deviation of 5.15 and 5.34 points, respectively (see Supplementary Figure S1). The mean of the VAS Wound questions ranged between 92.3 and 94.5 for the focus, between 51.6 and 56.7 for realism, and between 50.4 and 62.6 for unpleasantness (see Figure 3; Supplementary Table S1).

### Personality characteristics

#### Temperament and character inventory

Table 1 shows the TCI characteristics for the included study participants and a norm dataset provided by Datec (Leiden, the Netherlands). A student's *t*-test demonstrated that the study participants in our study showed different characteristics when compared to the norm group on three TCI characteristics: study participants showed lower scores for Harm avoidance [−2.9 (−5.7; −0.1) *p* = .04] and Self-Transcendence [−3.0 (−5.7; −0.3) *p* = .03], and higher scores for Novelty Seeking [3.5 (1.0; 6.0) *p* = .006] and Persistence [1.1 (0.3; 1.9) *p* = .0087]. Identified differences for healthy volunteers compared to the chronic pain group are in general for the same personality characteristics: Harm Avoidance [−9.1 (−12.4; −5.8) *p* < .001], Novelty Seeking [4.3 (1.7; 6.9) *p* = .0015], Persistence [1.4 (0.6; 2.2) *p* = .0005], Self-Directedness [4.3 (0.7; 7.9) *p* = .0185].

**Table 1:** Mean (SD) of Temperament and character inventory (TCI) for the study participants, norm group (data provided by datec) and chronic pain patients [data from Conrad et al. (25)].

	Study participants (*N* = 24)	Norm group (*N* = 167)	Chronic pain patients (*N* = 207)
Harm avoidance	9.6 (6.5)	12.5* (6.5)	18.7* (7.8)
Novelty seeking	22.5 (6.3)	19.0* (5.7)	18.2* (6.1)
Reward dependence	16.0 (4.1)	15.0 (3.8)	14.6 (4.2)
Persistence	5.6 (1.9)	4.5* (1.9)	4.2* (1.8)
Cooperativeness	32.2 (4.2)	32.4 (6.2)	30.6 (6.7)
Self-directedness	33.8 (5.0)	32.5 (7.0)	29.5* (8.7)
Self-transcendence	9.2 (4.5)	12.2* (6.5)	10.6 (5.6)

The asterisk (*) indicates a significant difference (*p* < .05) between groups.

#### Pain catastrophizing scale

Supplementary Table S2 shows an overview of the PCS results. Study participants scored on average 14.1 points (SD = 7.2), the lowest score was 0 and the highest score was 28.

#### Pain-anxiety symptoms scale

Supplementary Table S3 contains the overview of the PASS-20 results. The average total score of the PASS-20 questionnaire for all study participants was 27.1 (SD = 14.0), the lowest total score was 3 and the highest total score was 52.

#### Spielberger state-trait anxiety inventory

Summary data of the Spielberger Trait/Stage Anxiety Inventory is added to the supplement in Supplementary Table S4. On the trait questionnaire (STAI-DY2), study participants had a mean score of 50.2 (SD: 4.3).

The mean STAI-DY1 total score was slightly lower in the second visit (27.2) compared to the first (29.9). However, this difference was not statistically significant [95%CI: −2.7 (−0.1, 5.5) *p* = .055].

## Discussion

Here, we present the results of a study where VR was used to modulate the pain experience during a pain task. We demonstrate that VR can be used to enhance pain in the context of an evoked pain test. By introducing a virtual wound on the location of a painful stimulus, the PDT was lowered when compared to a neutral VR condition. Additionally, we demonstrated that study participants experienced the electrical stimulation as more unpleasant and more intense during the wound simulation, while the electrical simulation paradigm remained identical.

### Effects of virtual reality

A difference in PDT between the pre-VR neutral and VR neutral conditions was observed (see Figure 2). The PDT during the VR neutral condition was significantly higher than the PDT during the pre-VR neutral condition, suggesting a higher pain tolerability in the VR environment. Such effects were reported in previous studies as well, where, for example, wound treatment was perceived as less painful in VR than outside VR (26). Interestingly, in our study no differences were observed for perceived pain intensity and unpleasantness for the VR-neutral condition compared to the pre-VR neutral measurement. This might be caused by the relatively low number of assessments and the relatively high intra-subject variability. In conclusion, the immersion into (non-wound simulating) VR can be considered to increase pain detection thresholds.

The VR Wound condition resulted in lower pain detection thresholds compared to the neutral VR simulation. Additionally, the perceived pain intensity and unpleasantness were increased during the VR Wound condition. Combined, these observations indicate enhanced pain perception when immersed into a VR condition simulating a wound and thereby intensifying the stimulation by adding an affective component to the painful stimulus. Interestingly, the effect was more robust during the second visit during which the VR Wound condition was the first VR condition tested on that day. This suggests that there might be an effect of the order of VR conditions or stronger responses for the first assessment of a visit. We could not confirm this hypothesis due to the limited number of visits and the chosen order of the measurements. Nonetheless, the effect was overall large enough to allow us to demonstrate a statistically significant enhancement of the pain experience with the VR Wound condition.

We found no effect of VR simulation (i.e., VR Neutral vs. VR Wound) on the embodiment score (Supplementary Figure S1). Others suggested that the level of virtual body ownership could be considered a confounder when differences in outcomes exist (13, 14). However, study participants had a similar perception of body ownership in both the neutral and wound condition. The most likely explanation for this finding is that we used an extensive priming procedure in all VR simulations. The embodiment score was in general quite low and might be improved when the avatar can be more customized to the study participants or with a longer priming session before the measurements.

As mentioned earlier, few studies have adopted a similar approach to studying the effect of a simulation on the location of evoked pain in healthy volunteers (17, 18). These studies were both not executed in the settings typically used in drug development, but did show similar direction of results in lowering the PDT. In early drug development, repeated measures and assessments of concentration effects over the course of the day, as implemented in this study, are the standard. By testing this paradigm in these conditions with similar results, it becomes more feasible to use such a task in early drug development. Additionally, both studies did not include any questionnaires on pain experience, limiting the possibility to relate the findings to the affective component and pain experience.

The aim of this study was to add an affective (-motivational) component to a nociceptive stimulus to create a task more prone to respond to (dug induced) changes in the affective component of pain. Other studies often conclude that untangling the different domains of pain is not possible (4). If this conclusion holds true, experimental settings may lack sensations, emotions and cognitive processes due to their controlled laboratory nature. With this setup, we aimed to capture more dimensions of pain, including the affective dimension. Talbot recommends that future studies should ensure blinding of all involved and clear instructions for the study participants to prevent unintentional biases in questionnaire responses—a practice we also advocate based on our findings.

### Generalisation to chronic pain patients

This study shows how the pain experience during a pain task can be enhanced in healthy study participants, however how this relates to people with chronic pain is unclear. Because personality traits are often related to pain responses (27), we determined these in the study study participants in this study. This comparison provides information on the ecological validity of this study. The study participants in this study show significantly different personality traits on the TCI questionnaire compared to the norm group (Table 1). Significantly lower scores are found for HA and ST, and higher scores for NS. Other studies have already demonstrated that people with chronic pain are different from a normal population, with higher scores for HA and lower scores for NS, SD, and Cooperativeness (CO) (25). It therefore is possible that in a population with personality characteristics that are more similar to people with chronic pain, the effects of the pain test enhanced with VR may be different.

This study aimed to modulate the response on a painful stimulus possibly resulting in a challenge model for pain which includes an affective component. When properly validated, such a model could yield a biomarker that can be used in healthy study participants for early proof of concept of analgesic drugs aiming to reduce the affective component of pain. An early proof of concept in drug development can provide more insight in possible applications and patient stratification for future studies. Drugs that influence pain processing on a more central level may be beneficial for pain syndromes that currently remain untreatable (28, 29). A future study with the VR-pain setup will include an intervention to reduce the affective component of pain to provide the next step in validation. An example of such intervention could be an emotional altering drug, e.g., an anxiolytic.

### Study limitations

Due to the nature of the conditions, blinding of study participants was not possible in this study. As a result, we could not control for potential confounders including socially desirable responses. However, study participants were not told in advance which VR condition they would be presented with, and they were not informed on the hypothesis of the wound simulation. Future studies are recommended to include procedures that allow some way of blinding. For example, assessing the effect of medication on VR can be performed in a (double-) blind fashion in a crossover study allowing for a balanced number of VR neutral and VR wound assessments. Additionally, changing some of the stimulation procedures may aid in limiting anticipation effects (e.g., varying the rate of electrical stimulation).

**Figure 4:**
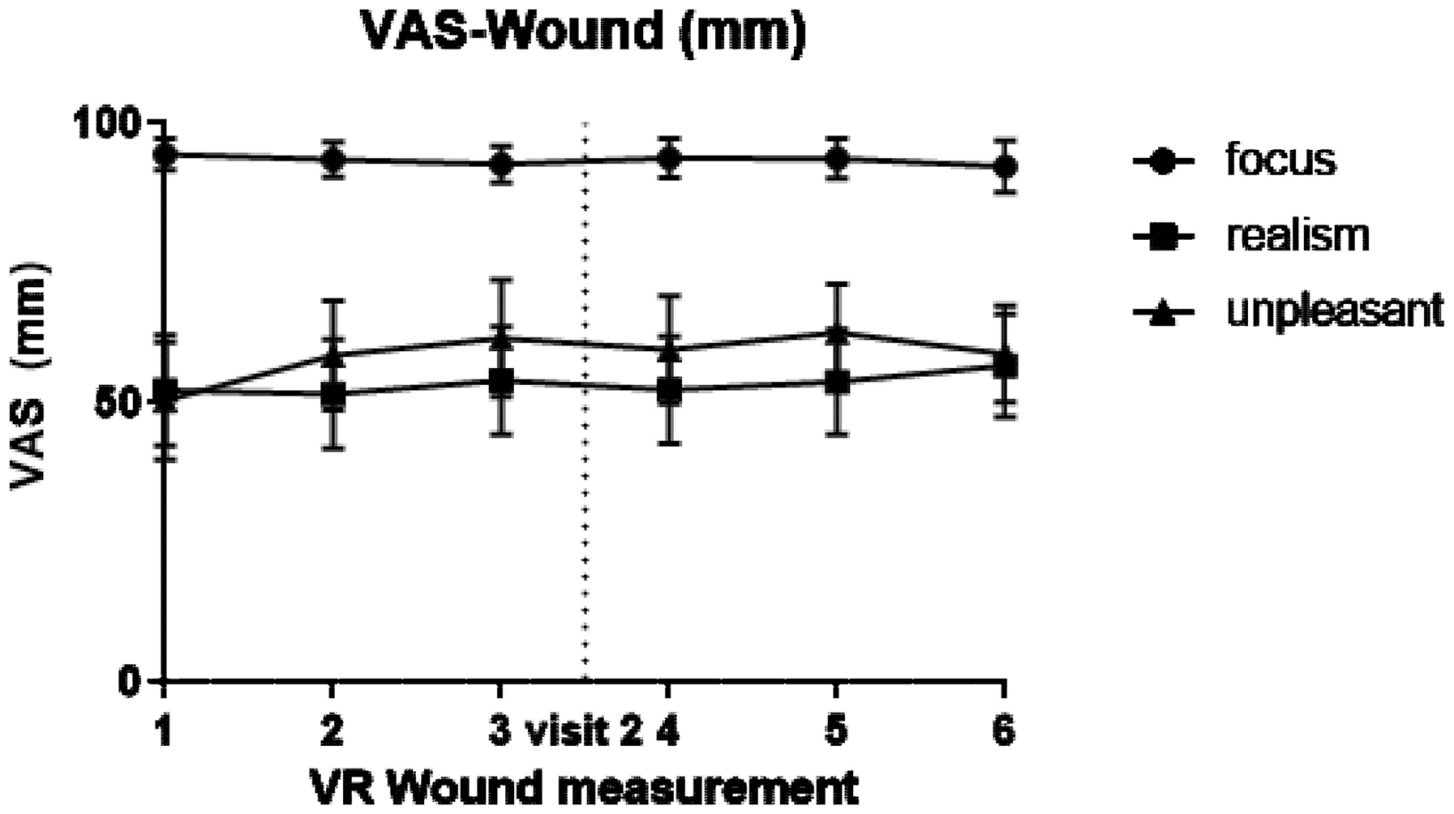
Graphical overview of visual analogue scale (VAS) scores regarding the virtual wound: focus, realism and unpleasantness.

All visual enhancements and progression of the wound were identical for each session with the VR wound simulation. However, unpredicted painful stimuli were experienced as more painful in an earlier study (8). It is therefore possible that repeated confrontation with the virtual wound would reduce the impact of the simulation. However, study participants reported consistent focus, realism, and unpleasantness throughout the session (see Figure 4). None of the three parameters of the VAS Wound questionnaire (i.e., focus, realism, and unpleasantness) showed significant variation over the visits or the different measurements. Also, the focus parameter indicates that study participants followed the instructions to look at the wound most of the time. Validation of this parameter can be done in future studies by including eye tracking in the VR setup and, for example, creating a heat map of visual focus.

To avoid the uncanny valley (relation between human likeness and a viewer's affinity toward it), a photo realistic wound was avoided. This resulted in the rather low (but stable) realism score. It can be imagined that different (possible improved) results may be obtained with a more realistic version of a wound or a simulation that has a better fit with the specific feeling of this test. A specific study aimed at determination of the optimal simulation may be considered for future research.

## Conclusion

This study is the first demonstrating the potential of VR in combination with a pain task to provide a challenge model highlighting the affective component of pain in a setting used in early phase drug development. The perceived level of immersion in the VR simulation was stable throughout the study making this setup feasible to use in drug studies with multiple visits and multiple measurements per day. Future studies should aim at validation for the use of proof of concept in early drug development.

## Data Availability

The original contributions presented in the study are included in the article/Supplementary Material, further inquiries can be directed to the corresponding author.
