# Supplementary material for: The impact of a virtual wound on pain sensitivity: insights into the affective dimension of pain
Source: Front Pain Res (Lausanne). 2025 Feb 26;6:1502616. doi: 10.3389/fpain.2025.1502616 (PMC11897489; doi:10.3389/fpain.2025.1502616)
Supplement: Supplementary file 1 [file Datasheet1.pdf]

Supplementary information

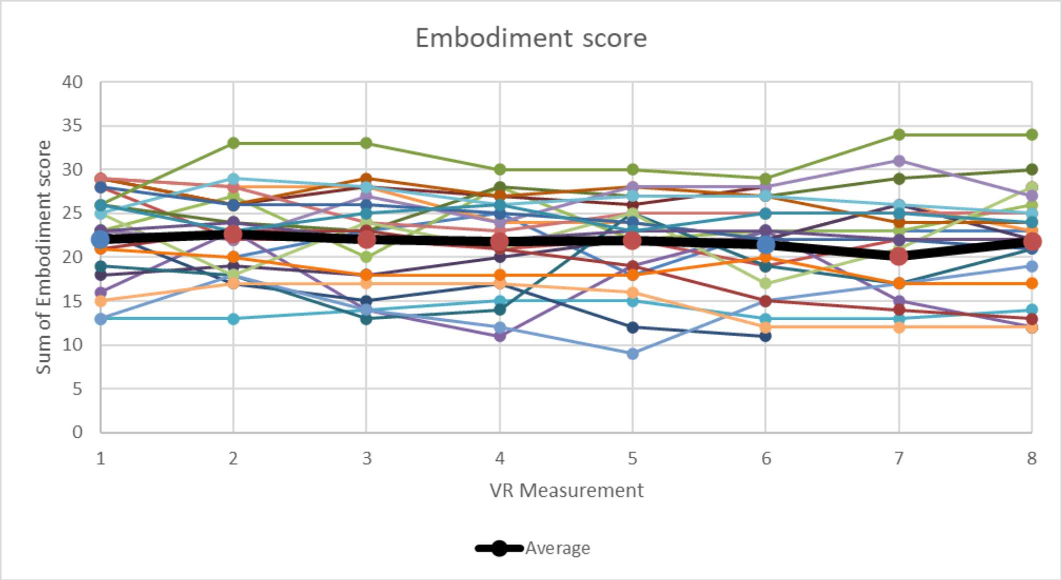

Figure 1 Sum of individual embodiment scores for all pain assessments including Virtual Reality. Black line indicates the mean, blue dots on this line the neutral simulation and red dots the wound simulation.

Table 1: Result of the Visual Analogue Scale (VAS) questions regarding the virtual wound. The amount of focus on the wound during the assessment, how realistic the wound was, and how unpleasant the wound simulation was experienced.

	FOCUS (+/-SD)	REALISM (+/-SD)	UNPLEASANT (+/-SD)
**VISIT 1 M1**	94.46 (+/-6.63)	52.25 (+/- 23.61)	50.42 (+/- 25.02)
**VISIT 1 M2**	93.54 (+/-7.28)	51.63 (+/- 23.11)	58.54 (+/- 23.07)
**VISIT 1 M3**	92.67 (+/-7.85)	54.08 (+/-22.73)	61.63 (+/- 24.62)
**VISIT 2 M1**	93.83 (+/-8.35)	52.38 (+/- 22.74)	59.54 (+/- 22.75)
**VISIT 2 M2**	93.73 (+/-8.16)	53.77 (+/- 21.10)	62.64 (+/- 19.59)
**VISIT 2 M3**	92.25 (+/-11.16)	56.71 (+/- 21.76)	58.79 (+/- 20.59)

Table 2 Pain Catastrophizing Scale

If I experience pain	MEAN	SD	RANGE
I worry about whether my pain will end	1.1	0.9	0 – 3
I feel I can't go on because of my pain	0.9	0.9	0 – 3
I think my pain is never going to get any better	0.5	0.7	0 – 2
I feel that my pain is overwhelming me	0.5	0.7	0 – 2
I feel I can't stand my pain anymore	1.0	0.8	0 – 3
I'm afraid that my pain will get worse	1.3	1.0	0 – 3
I keep thinking of other painful events	0.9	1.1	0 – 3
I anxiously want my pain to go away	2.0	1.0	0 – 4
I can't seem to keep my pain out of my mind	1.2	0.8	0 – 3
I keep thinking about how much I hurt	1.1	0.8	0 – 3

I keep thinking about how badly I want my pain to stop	1.5	1.1	0 – 3
I feel there is nothing I can do to reduce the intensity of my pain	1.0	0.9	0 – 3
I wonder whether something serious might happen	1.1	1.1	0 – 3
Sum score	14.1	7.2	0 – 28

Table 3 PASS-20

PASS-20 ITEM	MEAN	SD	RANGE
I think that if my pain gets too severe, it will never decrease	0.8	0.9	0 - 3
When I feel pain, I am afraid that something terrible will happen	0.9	1.0	0 - 3
I go immediately to bed when I feel severe pain	1.0	1.3	0 - 4
I begin trembling when engaged in activity that increases pain	0.8	0.8	0 - 3
I can't think straight when I am in pain	2.3	1.4	0 - 4
I will stop any activity as soon as I sense pain coming on	1.6	1.3	0 - 5
Pain seems to cause my heart to pound or race	1.4	1.2	0 - 4
As soon as pain comes on, I take medication to reduce it	1.2	1.3	0 - 5
When I feel pain, I think that I may be seriously ill	1.4	1.3	0 - 4
During painful episodes, it is difficult for me to think of anything else	1.5	1.1	0 - 4
I avoid important activities when hurt	1.7	1.2	0 - 4
When I sense pain I feel dizzy or faint	0.9	0.9	0 - 3
Pain sensations are terrifying	1.2	1.0	0 - 3
When I hurt I think about the pain constantly	1.8	1.2	0 - 4
Pain makes me nauseous	0.8	1.1	0 - 3
When pain comes on strong I think I might become paralyzed	0.6	1.1	0 - 3
I find it hard to concentrate when I hurt	2.4	1.1	1 - 5
I find it difficult to calm my body down after periods of pain	1.5	1.2	0 - 4
I worry when I am in pain	1.9	1.2	0 - 4
I try to avoid activities that cause pain	2.1	1.6	0 - 5
Sum score	27.1	14.0	3 - 52

Table 4: STAI-trait and state results sum of questions recorded during both study visits and screening.

	STAI-TRAIT MEAN (SD)	STAI-STATE MEAN (SD)
SCREENING	50.2 (4.3)	28.9 (6.0)
VISIT 1	-	29.9 (6.1)
VISIT 2	-	27.2 (5.4)
